# Using Oxygen Plasma Pretreatment to Enhance the Properties of F-Doped ZnO Films Prepared on Polyimide Substrates

**DOI:** 10.3390/ma11091501

**Published:** 2018-08-22

**Authors:** Chih-Cheng Chen, Fang-Hsing Wang, Sheng-Cheng Chang, Cheng-Fu Yang

**Affiliations:** 1 School of Information Engineering, Jimei University, Xiamen 361021, China; 201761000018@jmu.edu.cn; 2 Graduate Institute of Optoelectronic Engineering, National Chung Hsing University, Taichung 402, Taiwan; alex08156@hotmail.com; 3 Department of Chemical and Materials Engineering, National University of Kaohsiung, Kaohsiung 811, Taiwan

**Keywords:** oxygen (O_2_) plasma, pretreatment, F-doped ZnO, polyimide (PI) substrate

## Abstract

In this study, a radio frequency magnetron sputtering process was used to deposit F-doped ZnO (FZO) films on polyimide (PI) substrates. The thermal expansion effect of PI substrates induces distortion and bending, causing FZO films to peel and their electrical properties and crystallinity to deteriorate. To address these shortcomings, oxygen (O_2_) plasma was used to pretreat the surface of PI substrates using a plasma-enhanced chemical vapor deposition system before the FZO films were deposited. The effects of O_2_ plasma pretreatment time on the surface water contact angle, surface morphologies, and optical properties of the PI substrates were investigated. As the pretreatment time increased, so did the roughness of the PI substrates. After the FZO films had been deposited on the PI substrates, variations in the surface morphologies, crystalline structure, composition, electrical properties, and optical properties were investigated as a function of the O_2_ plasma pretreatment time. When this was 30 s, the FZO films had optimal optical and electrical properties. The resistivity was 3.153 × 10^−3^ Ω-cm, and the transmittance ratios of all films were greater than 90%. The X-ray photoelectron spectroscopy spectra of the FZO films, particularly the peaks for O_1s_, Zn 2p_1/2_, and Zn 2p_3/2_, were determined for films with O_2_ plasma pretreatment times of 0 and 30 s. Finally, a HCl solution was used to etch the surfaces of the deposited FZO films, and silicon-based thin-film solar cells were fabricated on the FZO/PI substrates. The effect of O_2_-plasma pretreatment time on the properties of the fabricated solar cells is thoroughly discussed.

## 1. Introduction

Transparent conducting oxides (TCOs) are electrically conductive, transparent oxide-based materials prepared via thin-film technologies and widely used in various optoelectronic devices, such as solar cells and flat panel displays (FPDs) [1]. In the past, TCO films based on a combination of indium (In) and tin (Sn) oxide have been widely used in applications of various optoelectronic devices due to their excellent conductivity and transparency. Because In metal is costly, TCO films based on the more reasonably priced zinc oxide (ZnO)—for example, undoped ZnO films [2], Al-doped ZnO (AZO) films [3], and F-doped ZnO (FZO) films [4]—have attracted much more interest because they have stable properties even under hydrogen plasma. N-type TCOs are of special importance for thin-film solar cell production because they are potential candidates for solar cell technology applications based on thin-film silicon.

Flexible optoelectronics have the advantages of light weight, superior flexibility, design variability, and low cost, so they are being investigated as next-generation optoelectronic devices for uses such as flexible displays, thin-film transistors, and thin-film solar cells [5]. To fabricate inexpensive, high-performance flexible optoelectronic devices, it is necessary to prepare TCOs with low resistance (≈300 Ω/□ or less for flexible liquid-crystal displays (LCDs), ≈100 Ω/□ for flexible organic light-emitting diodes) and a high transmittance ratio (>80%). Specially designed roll-to-roll (RTR) sputtering has been investigated as a coating technique to achieve high-efficiency TCO electrodes on flexible polyethylene terephthalate (PET) substrates. Past research has found that cost-efficient production of flexible TCO electrodes can be realized using a continuous RTR sputtering process. For example, Park et al. investigated the cost-efficient production of flexible indium-doped zinc oxide electrodes using continuous RTR sputtering [6].

Although TCO films on polymer substrates offer many advantages, they also are inherently brittle and bend under thermal tension, causing them to peel away from their polymer substrates and reducing their electrical properties [7]. The polymers also have a lower surface energy than the films, so the atoms on the polymer surfaces do not easily form new bonds with other atoms. Hence, TCO films have poorer adhesion on polymer substrates than on glass substrates [8,9]. Kim et al. found that when an O_2_ plasma pretreatment process was used prior to gallium-doped zinc oxide (GZO) sputtering, the surface energy and adhesion of the PET substrate improved, the contact angle decreased, and the root mean square (RMS) surface roughness increased significantly [10]. Kwon et al. treated a PI substrate with highly dense inductively-coupled O_2_ plasma from 60 to 600 s and found that the GZO films on PI substrates pretreated for 60 s had optimal crystallinity and minimal resistivity [8]. These results suggest that O_2_ plasma pretreatment could enhance adhesion between deposition films and PI substrates, so deposited TCO films would retain better electrical properties.

In this study, we used O_2_ plasma to modify the surfaces of PI substrates in an attempt to enhance their surface energy and prevent deterioration of their crystallinity and electrical properties. Previously, we investigated fluorine-doped ZnO (FZO) films grown by radio frequency (RF) sputtering on glass. These had high conductivity and transparency in the visible spectral range and could be widely employed as transparent electrodes in optoelectronic devices [4]. A HCl acid solution with a concentration of 0.2% was used to etch the surfaces of the FZO films to increase the haze ratio, which has been proven to increase the efficiency of fabricating p-i-n α-Si:H thin-film solar cells [11]. In our present study, we used a plasma-enhanced chemical vapor deposition (PECVD) system for O_2_ plasma pretreatment on PI substrates. After analyzing the contact angle and proving there had been an increase in the surface energy, we deposited FZO films on the PI substrates using an RF sputtering method. Once we had determined the optimal electrical properties, we deposited FZO films with a thickness of about 1000 nm, then etched the films to a depth of about 800 nm. From the spectra of the total and normal transmittances and the haze ratios of the FZO films, we found that etching yielded a higher haze ratio. Finally, we grew superstrate p-i-n α-Si:H thin-film solar cells using a single-chamber PECVD unit at 200 °C on the etched FZO/PI substrates and investigated their efficiency.

## 2. Experimental Procedure

F-doped ZnO powder was synthesized through a solid-state reaction method. Stoichiometric amounts of ZnF_2_ (purity 99.995%) and ZnO powder (purity 99.999%) were weighed according to the composition formula 1.5 wt% ZnF_2_ + ZnO. After the powders were mixed in acetone, dried, and ground, the solid-state reaction method was used to heat the F-doped ZnO powder in an air atmosphere. After being dried and ground, the F-doped ZnO was calcined at 600 °C for 1 h, then ground again. Next, it was mixed with polyvinyl alcohol (PVA) as a binder, and the result was uniaxially pressed into pellets of 5 mm thickness and 54 mm diameter using a steel die. After debinding, the F-doped ZnO pellets were sintered at 1060 °C for 3 h to complete the synthesis. The PI substrates were cleaned using deionized water, acetone, and isopropyl alcohol. Then PECVD was used for the O_2_ plasma pretreatment. At first, the pressure was pumped to 1 × 10^−5^ Torr, then the working pressure was controlled at 400 mTorr. O_2_ was used to treat the PI substrate’s surface.

Contact angle was measured using an FTÅ-125 (First Ten Ångstroms, Portsmouth, VA, USA). Five measurements were taken, and their values were averaged. The contact angle was calculated using Equation (1):

θ = 2 tan^−1^ (2h/d) (1)

where θ is the contact angle, and h and d are the height and base diameter of the water drop. The thickness of the deposited films was measured using a spectroscopic ellipsometer (SEMF-1000), and the adhesion force was measured experimentally with an atomic force microscope in force calibration mode [12]. The transmittance spectrum was measured using a UV-visible spectrometer (Hitachi U-3300, Hitachi Jigh-Tech, Tokyo, Japan) in a measurement range of 300–800 nm.

RF magnetron sputtering was developed as a fabrication technique to study how PI substrates that had undergone different O_2_ plasma pretreatment times affected the physical and electrical properties of the FZO films. The O_2_ plasma pretreatment power and temperature were 50 W and room temperature, and each sample’s area was 2.5 × 2.5 cm^2^. The FZO films’ thicknesses were measured using a spectroscopic ellipsometer and confirmed by field emission scanning electron microscopy (FESEM), while the FZO films’ crystalline structure was identified by X-ray diffraction (XRD). The sheet resistance was measured using a four-point probe (RT-70/RG-5, Napson, Tokyo, Japan). Hall parameters were measured (Ecopia HMS-300, Bridge Technology, Chandler Heights, AZ, USA) to find the resistivity, carrier concentration, and mobility and to determine whether the semiconductor was n-type or p-type. An X-ray photoelectron spectroscope (XPS: PHI5000 Versaprobe/Scanning ESCA Microprobe, ULVAC-PHI, Kanagawa, Japan) was used to carry out quantitative and qualitative chemical analyses, which were then used to determine the chemical bonds and compositions of the elements Zn and O.

After the physical, optical, and electrical properties of the FZO films had been measured, the thicknesses of the FZO films on the PI substrates pretreated for 30 s were increased to about 1000 nm by controlling the deposition time. The surfaces of the FZO films were etched by wet etching performed in dilute HCl solution (0.2% in H_2_O) to yield textured FZO films. The thickness of the etched FZO films was around 800 nm, achieved by controlling the etching time. The spectra of the total and normal transmittances and the haze ratio of the FZO films were measured using a haze meter (NDH-2000, Nippon Denshoku, Tokyo, Japan). Superstrate p-i-n α-Si:H thin-film solar cells were grown using a single-chamber PECVD unit at 200 °C on the etched FZO/PI substrates. The working pressure was 700 × 10^−3^ Torr and the deposition power was 20 W. Details of the fabrication and measurements of the thin-film solar cells can be found in Reference [11].

## 3. Results and Discussion

**Figure 1:**
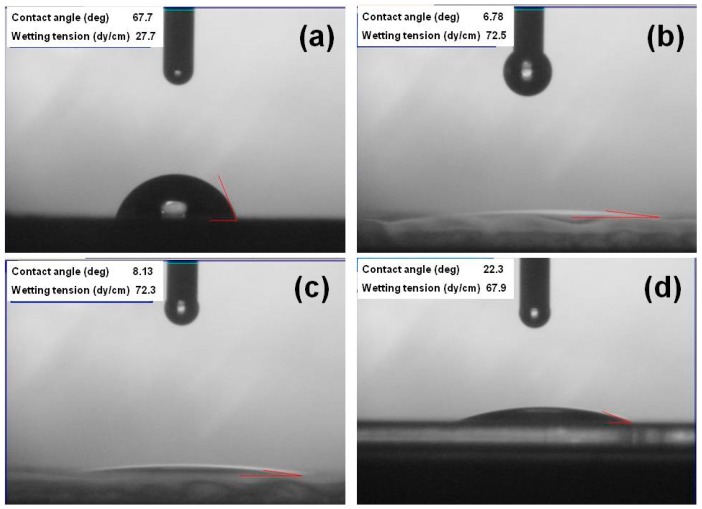
Contact angles of polyimide (PI) substrates with different O_2_ pretreatment times: (**a**) 0 s, (**b**) 60 s, (**c**) 120 s, and (**d**) 420 s.

The contact angles of PI substrates with O_2_ plasma pretreatment times of 0, 60, 120, and 420 s are compared in Figure 1. The contact angles for times of 0, 30, 60, 120, and 420 s were 67.7, 11.4, 6.78, 8.13, and 22.3, respectively. A smaller contact angle suggests a higher surface energy. Figure 1 demonstrates that the O_2_ plasma pretreatment increased the substrate’s surface energy. These results also indicate that the adhesive force on the PI substrates’ surfaces increased after O_2_ plasma pretreatment because a functional group formed on them and organic contaminants were removed from them [13]. Initially, the contact angle decreased significantly, reaching the lowest value when the pretreatment time was 60 s, then the angle slightly increased as the time rose from 60 to 420 s. The plasma pretreatment considerably enhanced the formation of an oxygen-containing polar functional group on the PI substrates’ surfaces. When a longer time was used (e.g., 420 s), the functional group’s activity slowed and the contact angle increased.

**Figure 2:**
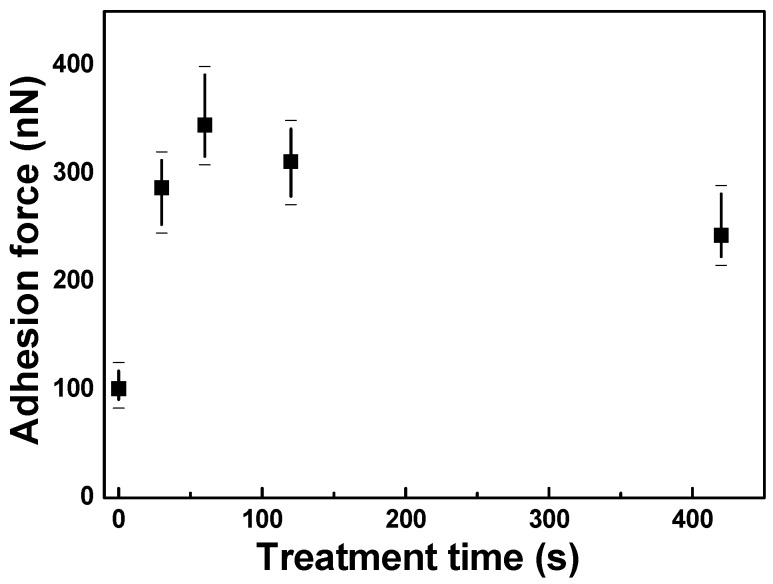
Adhesion force between F-doped ZnO (FZO) films and PI substrates as a function of O_2_ plasma pretreatment time.

Figure 2 shows the experimentally measured adhesion force between the FZO films and the PI substrates as a function of O_2_ plasma pretreatment time at a relative humidity of ≈30%. All of the FZO films showed a large standard deviation for the adhesion force. However, the change in adhesion force was similar to the change in contact angle. As O_2_ plasma pretreatment time increased, the adhesion force first increased, reaching a maximum at 120 s, then decreased as time was further extended. The low adhesion force of the PI substrate without O_2_ plasma pretreatment is attributed to the fact that without pretreatment, the substrate’s surface remained unmodified. The O_2_ plasma can modify the surfaces of polyimide substrates and enhance their surface energy, and then enhance their crystallinity and electrical properties.

**Figure 3:**
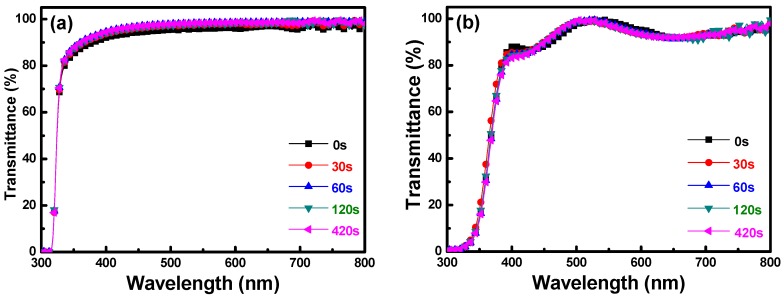
Transmittance spectra of (**a**) PI substrates and (**b**) FZO films on PI substrates as a function of O_2_ plasma pretreatment time.

Figure 3 shows the transmission spectra of the PI substrates and FZO films on the PI substrates, plotted against wavelengths in the region of 300–800 nm, with the O_2_ plasma pretreatment time as the parameter. As Figure 3a shows, when the times were 0, 30, 60, 120, and 420 s, the average transmittance ratio of the PI substrates was 95.5%, 96.2%, 96.9%, 96.6%, and 96.8%, respectively. The optical transmission from 400–800 nm was more than 82% for all FZO films, regardless of the O_2_ plasma pretreatment time. Figure 3b shows the transmission spectra of the FZO films on PI substrates as a function of O_2_ plasma pretreatment time. When the time was 0, 30, 60, 120, and 420 s, the average transmittance ratio of the FZO films was 93.7%, 93.3%, 93.3%, 93.3%, and 93.1%, respectively. In the transmission spectra of the PI substrates only and of the FZO films on PI substrates, as the time increased, the optical band edge experienced no apparent shift, but greater sharpness was noticeable in the curve of the absorption edge.

**Figure 4:**
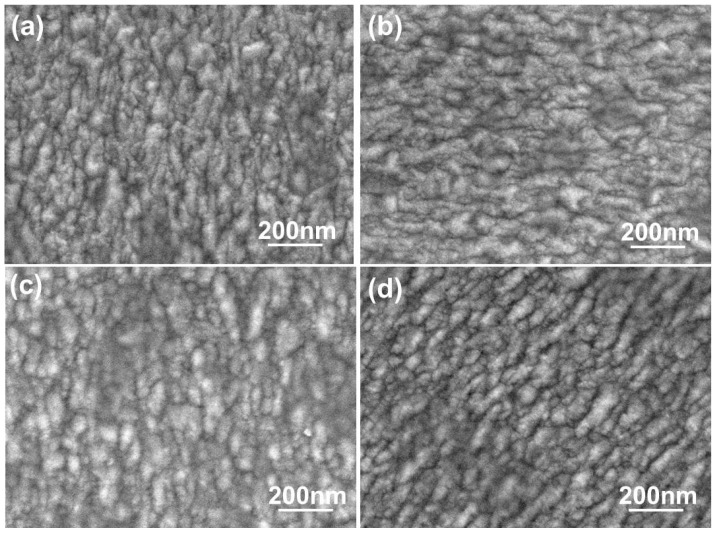
Surface SEM images of FZO films on PI substrates as a function of O_2_ plasma pretreatment time: (**a**) 0 s, (**b**) 30 s, (**c**) 60 s, and (**d**) 420 s.

The morphologies of the FZO films deposited on PI substrates as a function of O_2_ plasma pretreatment time are shown in Figure 4. The surface morphologies experienced no apparent change. When deposited at room temperature, the films had rough surfaces showing the nanocrystalline structure of the FZO grains. Figure 4 also shows that the grain-size distributions underwent no apparent change as the time increased, although average crystallite sizes were not easily calculated from surface observations. Nonetheless, these results suggest that the growth morphologies of the FZO films had no apparent effect on the electrical properties of the deposited FZO films.

**Figure 5:**
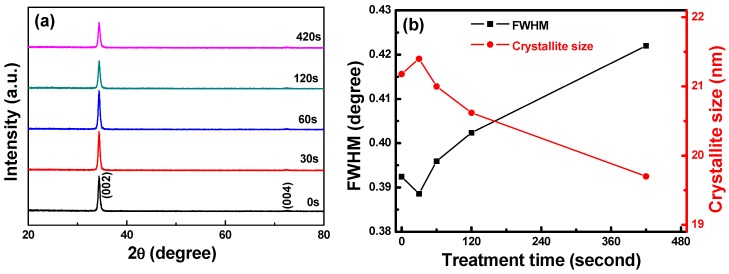
(**a**) XRD patterns and (**b**) full width at half maximum (FWHM) values and crystallite sizes of FZO films on PI substrates as a function of O_2_ plasma pretreatment time.

The XRD patterns of the FZO films on PI substrates are shown in Figure 5a as a function of O_2_ plasma pretreatment time. Only the diffraction peaks of the (002) plane and the unapparent (004) plane were observed. The diffraction intensity first increased as the time increased, reaching a maximum at 30 s, then decreased as the time was further increased. The full width at half maximum (FWHM) value of the (002) plane and the crystallite sizes of the FZO films on PI substrates are shown in Figure 5b as a function of O_2_ plasma pretreatment time. The 2θ value of the (002) plane remained unchanged at 34.36° as the time increased. The FWHM value of the (002) plane initially decreased, reaching a minimum when the pretreatment time was 30 s, then increased as the time was further increased. 

The Scherrer formula in Equation (2) can be used to find the crystallite sizes of the FZO films on the PI substrates:

D = (0.9 λ)/(β conθ)(2)

where D is the crystallite size, λ is 1.054056 nm, β is the FWHM value, and θ is the diffraction angle. The results in Figure 5b indicate that the FZO films deposited on 30 s pretreatment PI substrates had the largest crystallite sizes. However, Cullity and Stock found that as crystallite size increases, the grain boundary in the unit area decreases, the electronic mobility improves, and the resistivity drops [14]. The FZO films on 30 s PI substrates had the largest crystallite sizes, while the FWHM value increased and the diffraction intensity and crystallite size decreased. Hence, these films had the optimal electrical properties (minimum resistivity). When the time exceeded 30 s, the FWHM value increased and the diffraction intensity and crystallite size decreased, leading to poorer electrical properties, which will be proven in Figure 7.

The absorption coefficient α can be obtained using Equation (3), and the E_g_ value can be obtained using Equation (4):

*T* = (1 − R)^2^ exp(−αd) (3)

**Figure 6:**
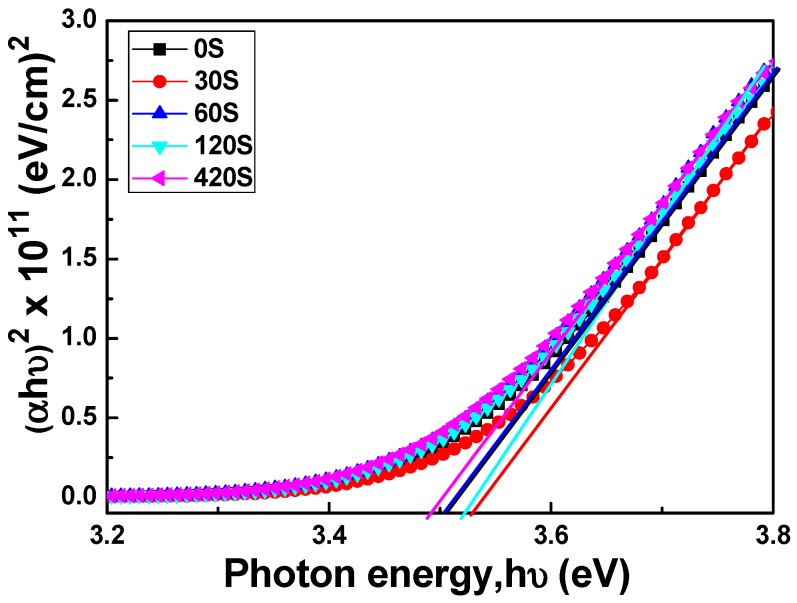
(αhν)^2^ versus hν − E_g_ of FZO films as a function of O_2_ plasma pretreatment time.

αhν = C (hν − E_g_)^1/2^ (4)

where T is transmittance, R is reflectance, d is film thickness, C is a constant, and hν is the incident light energy, respectively. Figure 6 plots (αhν)^2^ against hν (energy) in accordance with Equation (3), and the E_g_ values can be found by extrapolating a straight line at (αhν)^2^ = 0. The calculated E_g_ values of the FZO films as a function of pretreatment time can be obtained from the extrapolated straight lines in Figure 6. When the time was 0, 30, 60, 120, and 420 s, the E_g_ value of the FZO films on PI substrates was 3.505, 3.539, 3.501, 3.518, and 3.496 eV, respectively.

Many factors affect the transmission spectrum and thereby the E_g_ value. One factor is the carrier concentration of the FZO films. Figure 6 demonstrates that the E_g_ value varies in tandem with the carrier concentration. When the pretreatment times were 30 s and 120 s, the carrier concentrations were 2.242 × 10^20^ cm^−3^ and 2.152 × 10^20^ cm^−3^, respectively, corresponding to higher E_g_ values of 3.539 and 3.518 eV. Another factor is that the PI substrate pretreated for 30 s had the largest crystallite sizes and lowest FWHM value, and therefore fewer defects [15], so the FZO films on this substrate had a higher E_g_ value. 

**Figure 7:**
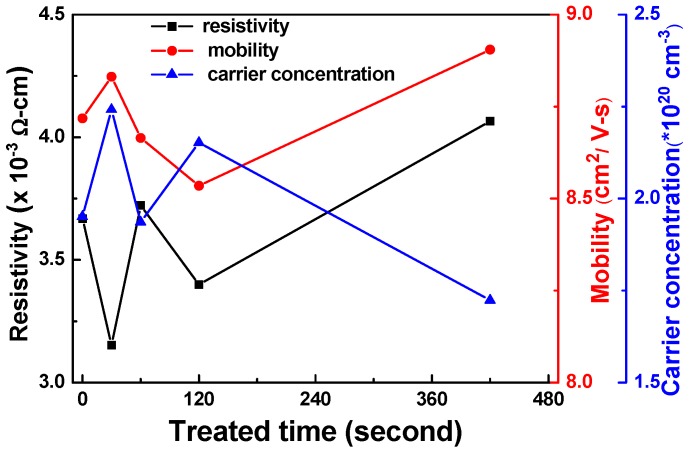
Hall measurement of FZO films as a function of O_2_ plasma pretreatment time.

Figure 7 presents Hall measurements of the FZO films for resistivity (ρ), Hall mobility (μ), and carrier concentration (n) as a function of O_2_ plasma pretreatment time. The results show that the Hall mobility and carrier concentration were enhanced when the pretreatment time was 30 s. These results indicate that the electrical characteristics of the FZO films were improved when the PI substrates were pretreated with O_2_ plasma; specifically, the surface energy and adhesive force on the PI substrate surfaces increased. Compared with substrates that had undergone no pretreatment, the Hall mobility and carrier concentration of the FZO films on 30 s PI substrates were enhanced by 1.5% and 14.9%, respectively, and the resistivity decreased from 3.669 × 10^−3^ Ω-cm to 3.153 × 10^−3^ Ω-cm. The electrical characteristics did not improve further when the pretreatment time exceeded 30 s. The XRD patterns in Figure 5a showed that a decrease in crystallinity lowered mobility and increased resistivity [16].

From Matthiessen’s Rule we know that total resistivity is affected by temperature, impurities, and defects, as expressed in Equation (5):

ρ (total) = ρ (thermal) + ρ (impurity) + ρ (defect)(5)

where ρ (total) is the total resistivity, ρ (thermal) is the resistivity caused by lattice vibration, ρ (impurity) is the resistivity caused by impurities, and ρ (defect) is the resistivity caused by defects in the deposited film. The XRD patterns in Figure 5a prove that PI substrates with a 30 s pretreatment time had better crystallinity. These results also suggest that O_2_ plasma pretreatment led to the formation of a functional group on the PI substrate surface and removed surface organic contaminants, changes that decreased the films’ contact angle, enhanced their adhesion and crystallinity, reduced their electronic scattering probability, and improved their conductivity (or decrease their resistivity).

**Figure 8:**
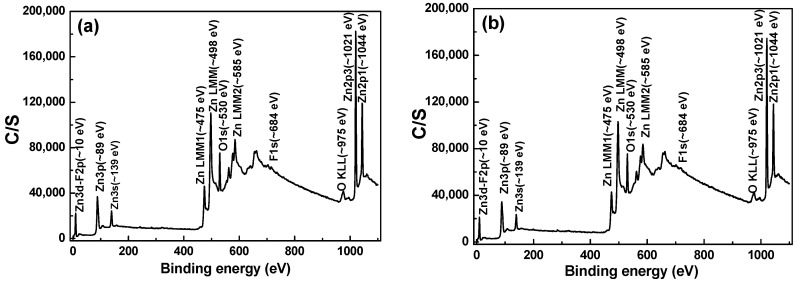
Full scanning X-ray photoelectron spectroscopy (XPS) spectra as a function of FZO films on different PI substrates: (**a**) Unmodified and (**b**) O_2_ plasma pretreatment time of 30 s.

The chemical structures of the FZO films on different PI substrates were investigated across the full scanning XPS spectra to clarify how the resistivity was improved. The XPS analyses of the PI substrates without pretreatment and with 60 s of pretreatment were measured and compared. We found that the intensities of the O_1s_ spectra were similar, weak, and difficult to analyze. In addition, for neither substrate could the Zn 2p_3/2_ and Zn 2p_1/2_ spectra be found (not shown here). As the results in Figure 8 show, the concentration of F^−^ ions was <0.1 at%, so the F_1s_ peak could not be analyzed. An O_1s_ peak at around 530.6 eV and Zn 2p_3/2_ and Zn 2p_1/2_ peaks at around 1021.2 and 1045.4 eV showed. No significant peak shift was observed in any of these as the pretreatment time increased from 0 to 30 s. As we know, bonding energies from 530 to 530.8 eV are found for the O_1s_ peak, while the Zn 2p_3/2_ peak is located from 1021.2 to 1022.2 eV and the Zn 2p_1/2_ peak from 1044.4 to 1045.4 eV. Hence, our analysis results are credible and fall within the range of values found in other studies.

**Figure 9:**
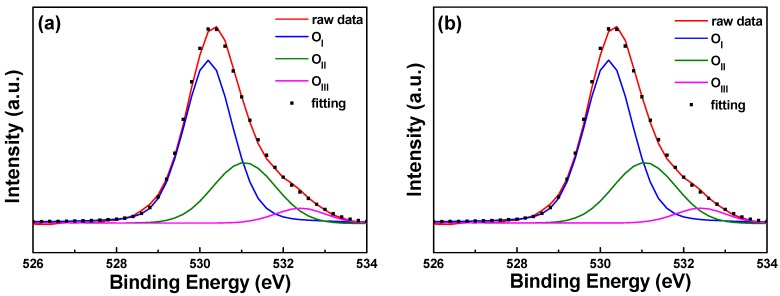
XPS spectra and the Gaussian-resolved components of the O_1s_ peak of FZO films on unmodified and 30 s pretreated PI substrates. (**a**) Unmodified and (**b**) O_2_ plasma pretreatment time of 30 s.

The typical surface O_1s_ peak could be consistently fitted by three nearly Gaussian curves, as shown in Figure 9. The peak was centered at 530.21 ± 0.12, 531.17 ± 0.18, and 532.41 ± 0.16 eV for the 0 s PI substrate and at 530.20 ± 0.11, 531.16 ± 0.16, and 532.4 eV ± 0.1 eV for the 30 s PI substrate. These results are similar to those obtained by Chen et al. for Al-doped ZnO films, which were centered at 530.2 ± 0.2, 531.1 ± 0, and 532.41 ± 0.16 eV in both as-deposited and annealed films [17]. The component of the O_1s_ spectrum at the low binding energy peak of 530.21 ± 0.12 eV is attributed to O^2−^ ions on the hexagonal wurtzite structure of a Zn^2+^ ion matrix, which are surrounded by Zn atoms (or substituted F atoms) with their full complement of nearest-neighbor O^2−^ ions [3]. The medium binding energy peak centered at 531.17 ± 0.18 eV is thought to be associated with O^2−^ ions in the oxygen-deficient regions within the ZnO matrix [11]. The high binding energy component located at 532.41 ± 0.1 eV is attributed to O_2_ absorbed on the chemical surfaces of the FZO films—for example, –CO_3_ and –OH and adsorbed H_2_O or O_2_ [18]. The XPS spectra of FZO films on unmodified and 30 s pretreated PI substrates are compared in Table 1; the area of the O_I_ peak decreased slightly and the areas of the O_II_ and O_III_ peaks increased slightly in the FZO films on the 30 s substrates.

**Figure 10:**
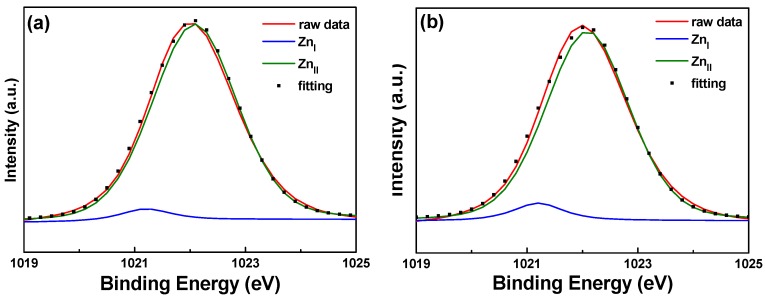
XPS spectra and the Gaussian-resolved components of the Zn 2p_3/2_ peak for FZO films on unmodified and 30 s pretreated PI substrates. (**a**) Unmodified and (**b**) O_2_ plasma pretreatment time of 30 s.

**Figure 11:**
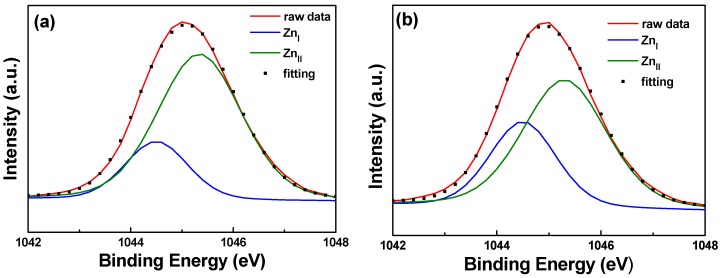
XPS spectra and the Gaussian-resolved components of the Zn 2p_1/2_ peak for FZO films on unmodified and 30 s pretreated PI substrates. (**a**) Unmodified and (**b**) O_2_ plasma pretreatment time of 30 s.

Figure 10 and Figure 11 show the XPS spectra and Gaussian-resolved components of the Zn 2p_3/2_ and Zn 2p_3/2_ peaks of FZO films on unmodified and 30 s pretreated PI substrates. As Figure 10 shows, the core line of the Zn 2p_3/2_ peak exhibits high symmetry in the FZO films, and the Zn 2p_3/2_ peak is separated into peaks at 1021.2 and 1022.2 eV. The low binding energy value of 1021.2 eV is caused by metal Zn, and only a small peak is observed, confirming that most of the Zn atoms existed in the oxidized state. The high binding energy value of 1022.2 eV indicates that the valence band of Zn^2+^ exists as the oxidization type within an oxygen-deficient ZnO_1−x_ matrix [18]. For both of the FZO films, the position of the 2p_3/2_ peak shows little variation with increases in O_2_ plasma pretreatment time (0 to 30 s), indicating the stable chemical state of Zn in the deposition films. The Zn 2p_1/2_ peak includes the metal Zn peak at 1044.4 eV and the Zn atoms in an oxidation state with a peak at 1045.4 eV, as Figure 11 shows. Comparison of the results in Figure 10 and Figure 11 indicates that in the XPS spectra of FZO films on unmodified and 30 s pretreated PI substrates, the area of the metal Zn (Zn_I_) peak increases and the area of the peak for atomic Zn (Zn_II_) in an oxidation state decreases. The areas of the Zn 2p_1/2_ and Zn 2p_3/2_ peaks for FZO films on unmodified and 30 s pretreated PI substrates are compared in Table 2.

**Figure 12:**
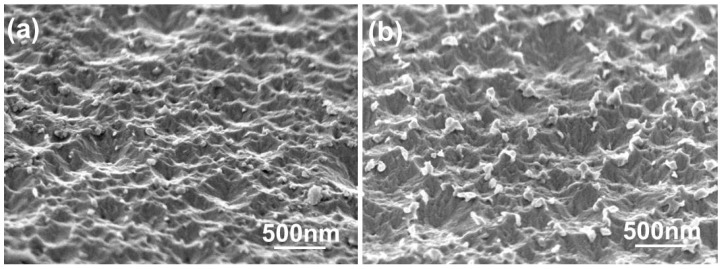
Surface morphologies of etched FZO films on (**a**) unmodified PI substrate and (**b**) PI substrate pretreated for 30 s.

Previously, we found that etched gallium-doped ZnO (GZO) films exhibited broad-band transparency and a high haze ratio, and that these films could be used as light-trapping structures in p-i-n α-Si:H thin-film solar cells [11]. The surface morphologies of the etched FZO films are shown in Figure 12. The PI substrates had 0 or 30 s of O_2_ plasma pretreatment, and the etching time was dependent on the etching rate. As the images in Figure 12 show, the surface roughness increased significantly after HCl etching, and the etching process gave the films’ surfaces a crater-like appearance.

**Figure 13:**
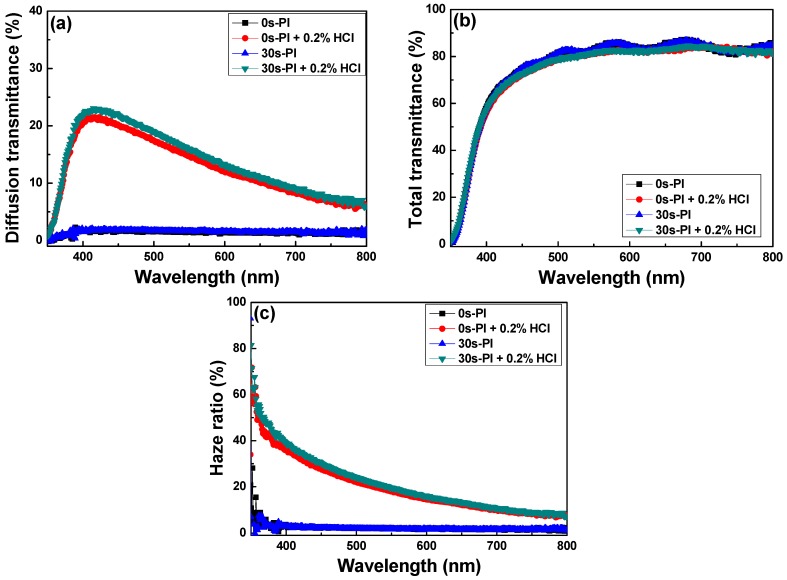
(**a**) Diffusion transmittance, (**b**) total transmittance, and (**c**) haze ratio of FZO films as functions of O_2_ plasma pretreatment time and HCl etching.

Figure 13 presents the total and diffuse transmittance spectra and the haze ratios of textured FZO films on different PI substrates. In general, diffuse transmittance is dependent on the surface structure and roughness of FZO films. As Figure 13a shows, the diffuse transmittances of the as-deposited FZO films in the visible light region (400–700 nm) were 1.12–1.97% and 1.31–2.07% when unmodified and 30 s pretreated PI substrates were used, whereas they experienced no apparent change when the light wavelength changed from 300 to 800 nm. When the HCl solution with a concentration of 0.2% was used to etch the FZO films, and the light wavelength was increased from 400 to 700 nm, the diffuse transmittances of the etched FZO films increased from 20.7% (at 400 nm) and 21.9% (at 400 nm) to a maximum of 21.5% (at 423 nm) and 23.0% (at 422 nm), then decreased to 8.32% (at 700 nm) and 9.10% (at 700 nm) as the unmodified and 30 s pretreated PI substrates were used. The average diffuse transmittances in the visible wavelength region varied from 1.49% to 14.9% (unmodified substrate) and from 1.63% to 16.1% (30 s pretreated substrate) as the etching process proceeded.

The total transmittance spectra of the FZO films are shown in Figure 13b as functions of O_2_ plasma pretreatment time and HCl etching. The average total transmittances of the as-deposited FZO thin-film substrates were 80.31% and 80.35% on the unmodified and 30 s pretreated PI substrates, whereas the textured FZO films showed that the average total transmittances decreased slightly to 78.62% and 78.27% in the visible (400–700 nm) wavelength region. These results suggest the etching process had no apparent effect on the average total transmittance, and the haze ratio is the important factor for the efficiency of the fabricated p-i-n α-Si:H thin-film solar cells. The ratio of diffuse transmittance to total transmittance is known as the haze ratio, and Figure 13c shows the haze ratio spectra of unetched and etched (textured) FZO films on different PI substrates. The haze ratios of the textured FZO thin films decreased with increasing light wavelength. The average haze ratios of the unetched FZO thin films in the visible wavelength region with unmodified or 30 s pretreated PI substrates were 1.88% and 2.06%, compared with 19.5% and 21.2% for the textured FZO films.

In the past, we found that increasing the haze ratio increases the efficiency of fabricated α-Si thin-film solar cells [11]. Because the HCl-etched FZO films had higher diffuse transmittance and haze ratios, they were used to fabricate α-Si thin-film solar cells. The mobility of electrons in α-Si:H is roughly one or two orders greater than that of holes, and the collection of electrons better than holes moving from p- to n-type contact. Hence, p-i-n superstrate hydrogenated α-Si thin-film solar cells were fabricated using a single-chamber PECVD unit at 200 °C. When etched FZO films on different PI substrates were used, the open-circuit voltage (Voc), short-circuit current density (Jsc), fill factor (F.F.), and efficiency (η) values were measured. 

**Figure 14:**
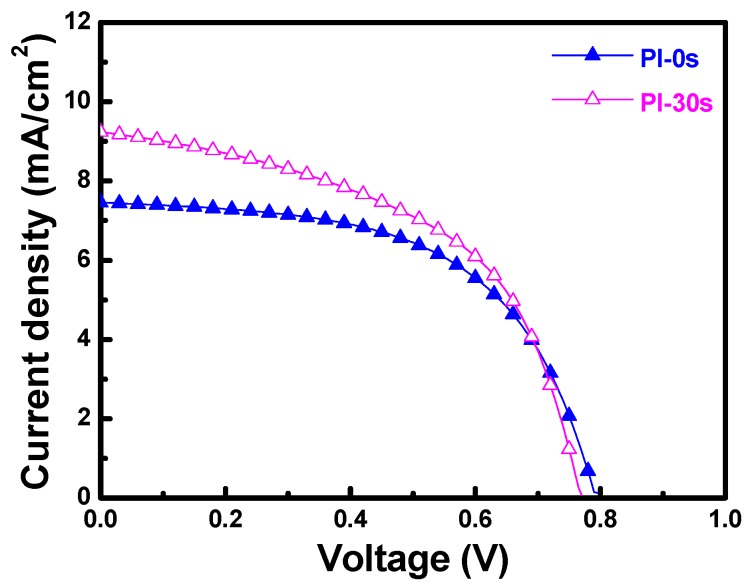
Current–voltage properties of fabricated p-i-n α-Si:H thin-film solar cells.

Figure 14 shows that when PI substrates with 0 and 30 s of pretreatment were used to fabricate the α-Si thin-film solar cells, the Voc values of the solar cells were 0.795 and 0.770 V, the Jsc values were 7.455 and 9.235 mA cm^−2^, the F.F. values were 0.566 and 0.518, and the efficiencies were 3.35 ± 0.12 and 3.68 ± 0.11, respectively. Apparently, the efficiency increased 9.4% when the 30 s pretreated substrate was used to fabricate the p-i-n α-Si:H thin-film solar cells. As Figure 14 shows, the Voc value and F.F. value decreased slightly, but the Jsc value and the efficiency increased when 30 s pretreated PI substrates were used. The results in Figure 13c indicate that the haze ratio of the etched FZO films on the 30 s pretreated PI substrates was lower than on the unmodified PI substrates. Hence, we know that although the haze ratio causes the increases in the Jsc value and efficiency of the fabricated α-Si thin-film solar cells, there is another contributing factor in the Jsc value as well. These results also prove that the O_2_ plasma pretreatment of the PI substrates increased the adhesive force and crystallization of the FZO films, so the resulting deposited FZO films had better electrical properties (see Figure 7). When the 30 s pretreated PI substrate was used to fabricate p-i-n α-Si:H thin-film solar cells, it caused increases in the Jsc value and efficiency of the fabricated α-Si thin-film solar cells. 

## 4. Conclusions

When the O_2_ plasma pretreatment times of the PI substrates were 0, 30, 60, 120, and 420 s, the contact angles were 68.6, 11.4, 6.39, 7.93, and 22.3, and the average transmittance ratios were 95.5, 96.2, 96.9, 96.6, and 96.8, respectively. The O_1s_ peak was centered at 530.21 ± 0.12, 531.17 ± 0.18, and 532.41 ± 0.16 eV in the 0 s PI substrate and at 530.20 ± 0.11, 531.16 ± 0.16, and 532.4 eV ± 0.1 eV in the 30 s PI substrate. The core line of the Zn 2p_3/2_ peak exhibited a high degree of symmetry in the FZO films, and the Zn 2p_3/2_ peak was separated into peaks at 1021.2 and 1022.2 eV. The Zn 2p_1/2_ peak included a metal Zn peak at 1044.4 eV and a Zn atom in an oxidation state with a peak at 1045.4 eV. For the FZO films, only the diffraction peaks of the (002) plane and the unapparent (004) plane were observed, and the diffraction intensity (FWHM value) of the (002) plane first increased (decreased) as the O_2_ plasma pretreatment time increased, reached a maximum (minimum) at 30 s, then decreased (increased) as the time was extended. When PI substrates pretreated for 0 s or 30 s were used to fabricate α-Si thin-film solar cells, the Voc values of the solar cells were 0.795 and 0.770 V, the Jsc values were 7.455 and 9.235 mA/cm^2^, the F.F. values were 0.566 and 0.518, and the efficiencies were 3.35 ± 0.12 and 3.68 ± 0.11, respectively. This study’s results have proven that O_2_ plasma pretreatment of the PI substrates increased the adhesive force and crystallization of the FZO films and that the etching process increased the haze ratio of the FZO films, causing an increase in the Jsc value and boosting the efficiency of the fabricated α-Si thin-film solar cells.

## Figures and Tables

**Table 1:** Area of the O_1s_ peak of F-doped ZnO (FZO) films on unmodified and 30 s pretreated polyimide (PI) substrates.

Treatment Time (s)	O_I_ Peak (%)	O_II_ Peak (%)	O_III_ Peak (%)
0	65.92	28.07	6.01
30	63.26	29.44	7.31

**Table 2:** Areas of the Zn 2p_1/2_ and Zn 2p_3/2_ peaks for FZO films on unmodified and 30 s pretreated PI substrates.

Treatment Time (s)	Zn 2p_1/2_		Zn 2p_3/2_	
	Zn_I_ Peak (%)	Zn_II_ Peak (%)	Zn_I_ Peak (%)	Zn_II_ Peak (%)
0	25.18	74.82	4.98	95.02
30	33.54	66.46	6.71	93.29
